# Compliance with a personalised home exercise programme in chronic low back pain patients after a multidisciplinary programme: A pilot randomised controlled trial

**DOI:** 10.3389/fresc.2022.1050157

**Published:** 2022-11-17

**Authors:** R. Lenoir dit Caron, M. Rouzée, J. Coquart, M. Gilliaux

**Affiliations:** ^1^ Normandie University, CETAPS Laboratory, UR 3832, Mont Saint Aignan, France; ^2^ Clinical Research Department, La Musse Hospital (Fondation La Renaissance Sanitaire), Saint-Sébastien-de-Morsent, France; ^3^ Cabinet de Kinésithérapie, Ferrière-Haut-Clocher, France; ^4^ Unité de Recherche Pluridisciplinaire Sport Santé Société” is a departement, Univ. Lille, Univ. Artois, Univ. Littoral Côte D'Opale, ULR 7369 - URePSSS - Unité de Recherche Pluridisciplinaire Sport Santé Société, Liévin, France

**Keywords:** rehabilitation, low back pain, exercise, international classification of functioning disability and health, treatment adherence and compliance

## Abstract

**Background:**

Chronic low back pain (CLBP) is a very common problem throughout the world. One treatment possibility is the multidisciplinary programme (MP) in a rehabilitation centre, which provides intensive rehabilitation through physical exercise to quickly improve the patient conditions. Patients nevertheless do not always continue the exercises when they return home. This study thus evaluated compliance with a personalised home-based programme for CLBP patients post-MP.

**Methods:**

A randomised controlled single-blind trial was conducted. Thirty patients were randomised into two groups and participated in an MP for 4 weeks. They were then given an exercise booklet for home rehabilitation. In addition, each patient in the experimental group constructed a personalised exercise programme with a physiotherapist. The control group was only encouraged to continue the exercises at home. To assess therapeutic compliance, both groups were asked to document each completed exercise in a logbook. In addition, pain intensity, flexibility, muscle endurance, activity limitations, participation restrictions, and beliefs about physical activity were assessed at the beginning and end of the MP and again after 12 weeks at home.

**Results:**

Compliance was good for all activities in both groups, but there were no significant differences between groups. All participants improved on the criteria by the end of MP, and both groups maintained the improvements in most of the criteria at 3-month follow-up.

**Conclusion:**

This study showed the effectiveness of an MP for CLBP in the short and medium term. However, future research should focus on longer-term compliance.

## Introduction

Chronic low back pain (CLBP) is a major public health problem that affects people of all ages and is a leading contributor to disease burden worldwide (1). It is defined as persistent pain in the lumbar region that has been present for more than 3 months, and it may radiate to the buttocks, iliac crest, and thigh, sometimes extending to the knee. The most common form is non-specific CLBP, which means without inflammatory, traumatic, tumoral or infectious origin. Non-specific CLBP does not have a known pathoanatomical cause, and its transition from the acute to the chronic phase is complex and depends on many individual, professional and psychosocial factors. This is probably why so many therapies are now addressing this health problem.

Treatment options for CLBP comprise physical therapies (massage, spinal manipulation, exercise), psychological therapies (cognitive behavioural therapy, acceptance and commitment therapy), and complementary therapies (mindfulness-based stress reduction, yoga, tai chi, acupuncture). When these usual types of management fail, the patient may be referred to a multidisciplinary programme. These programmes target the physical, psychological and social aspects of CLBP, involve a team of clinicians, and are recommended in several major international clinical guidelines such as the National Institute for Health and Care Excellence, the American College of Physicians, the German Disease Management Guidelines, and the French National Authority for Health (2, 3).

Numerous studies have shown the effectiveness of multidisciplinary programmes that deal with pain, functional disability, quality of life and the return to work (4–6). However, the benefits do not always seem to last over the long term (6, 7), and the lack of long-term benefits may be due to issues of patient compliance. As long as patients are in the multidisciplinary programme, they are guided in performing physical activities, supported by a professional team, stimulated by the other patients, and given access to several types of sports equipment. Hospitalisation and medical leave allow them to focus solely on their recovery. But when they return home, they no longer have access to the same equipment, the same professionals, or the stimulation of group activities. Moreover, the return to daily life and work no longer necessarily leaves them the time or the motivation to exercise, even though the persistence of long-term benefits seems to be closely linked to the regular practice of physical activity (8–10). A home exercise programme therefore seems relevant in this context. It would allow certain patients to finalise their rehabilitation and would accompany them towards self-management of their physical capital over the long term.

Hartigan et al. (2000) investigated the compliance of CLBP patients with physical activity after an intensive rehabilitation programme. After discharge from hospital, patients received individualised written recommendations for home exercise. The authors found a significant positive change in compliance between the initial follow-up assessment at 3 months and then again at 1 year, thus demonstrating that patients are indeed able to adhere to a personalised home exercise programme. However, the study did not have a control group (10). It therefore seemed important to carry out a randomised controlled trial evaluating the compliance with and effectiveness of a personalised home exercise programme following multidisciplinary rehabilitation.

The primary objective was to assess compliance with a personalised home exercise programme after multidisciplinary rehabilitation for CLBP. The secondary objective was to evaluate the effectiveness of a multidisciplinary programme according to the International Classification of Functioning, Disability and Health (ICF).

## Methods

### Study design

A single-blinded randomised controlled trial was performed in accordance with Consolidated Standards of Reporting Trials (CONSORT) criteria. The research was conducted in a French rehabilitation centre and approved by the independent protection committee Ouest VI (IDRCB number: 2017-A03656-47).

### Participants

All patients referred to the multidisciplinary programme between June and November 2018 were offered the possibility of participating. No sample calculation was done initially because this was a pilot study with a limited experimental period. Inclusion criteria were: age between 18 and 65 years, non-specific chronic low back pain, pain greater than 25 mm on a visual analogue scale on exertion, informed consent from the patient, affiliation with a health insurance system, and no participation in other studies during the experimental period. Exclusion criteria were: medical contraindication to the practice of physical activity, a psychological condition precluding participation in the exercises, insufficient mastery of the French language that would prevent understanding the instructions and evaluation questionnaires, and any surgical operation scheduled during the experimental period.

### Intervention

During the first phase of the experiment, all patients completed the same multidisciplinary programme over 4 weeks. This was considered a part-time hospitalisation, from 9 a.m. to 4 p.m., Monday to Friday. All patients were supervised by the same care team, which included: physical activity educators, a physiotherapist, an occupational therapist, a nurse, a psychologist, a social worker and a rehabilitation physician. Patients were divided into small groups (8 to 10 people) and engaged in activities supervised by various team members. The physical activity educators supervised daily physical therapies such as stretching, muscle strengthening (body weight and machine strength training), aerobic activity (walking or cycling), adapted sports (e.g. basketball, handball, ping-pong, badminton, tai chi) and balneotherapy. The physiotherapist provided individual physiotherapy sessions for cases of intense pain. The occupational therapist led educational workshops on how to manage back pain in everyday life (e.g., how to position yourself to carry a heavy weight), and the nurse delivered the medication prescribed by the physician and participated in the patient's pain education. The psychologist received patients individually if they wished to have psychotherapy sessions. The social worker accompanied them in their administrative procedures, particularly with regard to their work. The rehabilitation physician was in charge of patient inclusion, medical prescriptions, and supervised team meetings.

At the end of the 4 weeks of intensive rehabilitation, each participant was received for a one-to-one interview with an investigator (RL). During this interview, they each received a booklet with descriptions and illustrations of exercises they could practise at home, divided into three parts: stretching, muscle strengthening, and aerobic activities. All the exercises described in this booklet were familiar to them because they had practiced them during the multidisciplinary programme. The stretching part started with explanations on the benefits of stretching and general instructions (e.g., you must feel a stretching of your muscle but not an intense pain, you must hold the position 30 s etc.). Then for each muscle or muscle group several stretching positions were proposed with an illustration and a description. The target muscles were: hamstring, sural triceps, glutes, adductors, ilio-psoas, quadriceps, back stretch (flexion, extension, tilt, rotation), pectorals, triceps brachii, neck muscle. The muscle strengthening section also began with an explanation of the benefits followed by general instructions (e.g., customize the hold time, number of repetitions, etc.). The exercises target the stabilizing muscles of the trunk, with core-exercise essentially, but also strengthening of the lower limbs (e.g., squats, lunges) and the upper limbs (e.g., push-ups). For each exercise, different levels of difficulty were proposed. The section on aerobic activity began with a definition, explanation of the benefits and the difference between interval and endurance work. Several activities were given as examples: walking, swimming, cycling, stair climbing, fitness etc. Based on this booklet, they were all advised to do a minimum of 150 min of exercise per week and to vary the activities.

The experimental group also planned a weekly exercise programme adapted to their schedules and preferences, for 12 weeks. The guidelines for creating this programme were to include a minimum of 150 min of weekly exercise, with at least 30 min of each type of activity (stretching, muscle strengthening, aerobic activities). This personalised programme was in the form of a calendar and they were advised to place it in a visible spot (e.g., on the fridge door). It was a motivational and organisational support, not an obligation to perform the planned exercises. Thus, patients were free to rearrange this schedule by moving planned sessions or adding to it. The aim was to help the patients anticipate their return home and feel supported and advised in their organisational choices by a professional.

### Assessment procedure

The evaluators were a physical activity educator and a physiotherapist, blind to the participants' group allocation. They followed a standardised assessment protocol based on the ICF, and only the data on compliance was unknown to them. Patients were assessed three times by the same evaluator: before the multidisciplinary programme (T0), post-treatment (T1), and 3 months after their return home (T2).

Regarding our primary outcome, which was patient compliance, patients were asked to document their daily exercise in the logbook once they returned home, noting each day the nature of the activities performed (stretching, muscle strengthening, or aerobic activity) and the duration (in minutes). This logbook looked different for each group: for the control group it was completely blank and for the experimental group it contained the personalised home-based programme.

The assessment protocol for our secondary outcomes was based on the ICF in order to cover all components of a health problem like non-specific CLBP. Regarding physical impairments, the visual analogue scale (at rest and during activity, in millimetres) was used as a measure of pain intensity (11, 12), with a high score representing a high level of pain. The fingertip-to-floor and heel-to-buttock distances (in centimetres) were used to assess mobility of the lumbopelvic-femoral complex and the quadriceps, respectively; a smaller distance indicates better flexibility (13). Sorensen and Shirado tests (in seconds) were used to measure back and abdominal muscle endurance, respectively; a longer duration reflects better endurance (14–18). In order to assess activity limitations, the French version of the Roland Disability Questionnaire (24-point score) was used; the higher the score, the more subjects are limited in their daily activities (19). To assess participation restrictions, the subscales “Work and leisure activities”, “Anxiety/depression” and “Sociability” of the Dallas Pain Questionnaire (percentage) were selected (20). We removed the subscale on “Daily activities” because it was redundant with the Roland Disability Questionnaire. The higher the score, the more restricted the patient's participation. Finally, the physical subscale of the Fear-Avoidance Belief Questionnaire (18-point score) was used to assess patients' beliefs about physical activity (21). The last two questions of these questionnaires were difficult to understand, so we removed them. The higher the score, the more the participants thought that physical activity was harmful for their back.

### Randomisation

Randomisation was carried out using computer software by an investigator (MG) who had no contact with the participants. Minimization was performed to reduce the risk of differences between the groups on the pain intensity criterion. Indeed, minimization aims to ensure treatment arms are balanced with respect to predefined patient factors (22). For each patient included, a computer algorithm calculates in real time the allocation of the group that guarantees the best possible balance between the groups. The first patient is randomly allocated to a group. Then, for each additional patient, the allocation of the treatment is made in order to minimize the imbalance between the groups, considering the values of the patient stratification criteria and the patients already randomized. In this case, the stratification criteria was pain intensity during activity, measured with visual analogue scale. As pointed out by Mannion et al. (2007), this measure is probably the most sensitive to changes in the patient's health status regarding CLBP, is one of the best determinants of disability, and would therefore be closely related to whether or not the patient can perform exercise (12). Considering that a score below 25 mm on the visual analogue scale indicates acceptable pain intensity (see inclusion criteria), the aim is to obtain patients with scores between 0 and 25 mm. Thus, our stratification criterion was set at 62.5 mm, based on the following calculation: [(100 − 25)/2 + 25].

### Statistical analysis

Statistical analysis was performed with Sigmaplot 14.0 for Windows. Data were considered parametric if they were continuous and if normality and variance were confirmed. Data were considered non-parametric if they were ordinal or if they were continuous without normality or variance being met. Statistical significance was set at *p* < 0.05.

Regarding compliance for each type of activity, paired sample t-tests and Mann-Whitney tests were performed for the parametric and non-parametric data, respectively, in order to compare the time spent exercising and the ratios (exercise time achieved/exercise time recommended) between groups. To analyse the change in the time spent doing the three types of activities in each group over the 12 weeks of the personalised home programme, a one-factor (time factor) repeated measures analysis of variance (ANOVA) was applied for each variable (group and type of activity) for the parametric data. In cases of significant interaction, a Bonferroni post-hoc test was performed. For the non-parametric data, a Friedman test was performed, and in the case of significant interaction, a post-hoc Tukey test was conducted.

For the ICF-related variables, paired sample t-tests and Mann-Whitney tests were performed for the parametric and non-parametric data, respectively, to ensure that the groups were comparable at T0 and remained so at T1. Then, a two-way (time and group factors) repeated measures ANOVA was performed for the parametric data to evaluate the change in the ICF-related variables between T0 and T1, and between T1 and T2. A Bonferroni post-hoc test was performed in the case of significant results. For the non-parametric data, a Wilcoxon test was used for the intra-group analysis and a Mann-Whitney test was performed for the inter-group analysis.

## Results

### Sample

**Figure 1:**
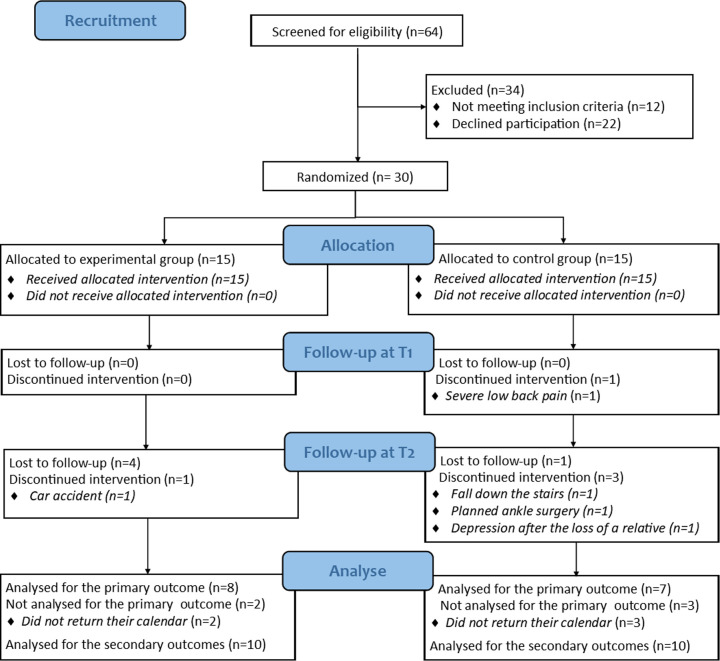
CONSORT participants flow diagram.

A convenience sample of 30 participants was recruited for this study, with 16 women and 14 men whose average age was 43.2 ± 11.0 years (Figure 1). The experimental and control groups had 15 patients each. One participant prematurely left the multidisciplinary programme because his back pain did not allow him to participate in the activities, but we do not know whether this decision was due to the programme itself. During the home-based programme (T1-T2), four participants (one in the experimental group and three in the control) had to stop doing their exercises: one fell on the stairs, one had a car accident, one underwent a scheduled ankle operation that had not been mentioned at inclusion, and one became depressed after the loss of a relative. These discontinued interventions were not related to the practice of the home exercise programme.

### Compliance with the home-based programme

The inter-group analysis of compliance (ratio = time achieved/time prescribed) and time spent performing the exercises over the 12 weeks showed no significant difference for stretching (*p* = 0.336), muscle strengthening (*p* = 0.536), aerobic activity (*p* = 0.976), or total exercise (*p* = 0.905) (Table 1).

**Table 1:** Exercise compliance over the 12-week home-based programme.

	Experimental (*n* = 8) Mean (SD)	Control (*n* = 7) Mean (SD)
**Ratio, %**		
Stretching	106.3 [81.3; 384.7]*	211.1 [168.1; 222.2]*
Muscle strengthening	68.8 [10.4; 190.3]*	98.6 [48.3; 188.1]*
Aerobic Activity	476.8 (412)	482 (412)
Total	99.5 (52.7)	96.3 (48.1)
**Sum, min**		
Stretching	382.5 [292.5; 1,385]*	760 [605; 800]*
Muscle strengthening	247.5 [37.5; 685]*	355 [174; 677]*
Aerobic Activity	1,716.6 (771.8)	1,735.3 (1,483.3)
Total	2,985.4 (1,579.5)	2,889.7 (1,443.5)

SD, standard deviation.

Significant results (*p* < 0.05) are in **bold**.

* Median [Quartile 1–Quartile 3].

**Figure 2:**
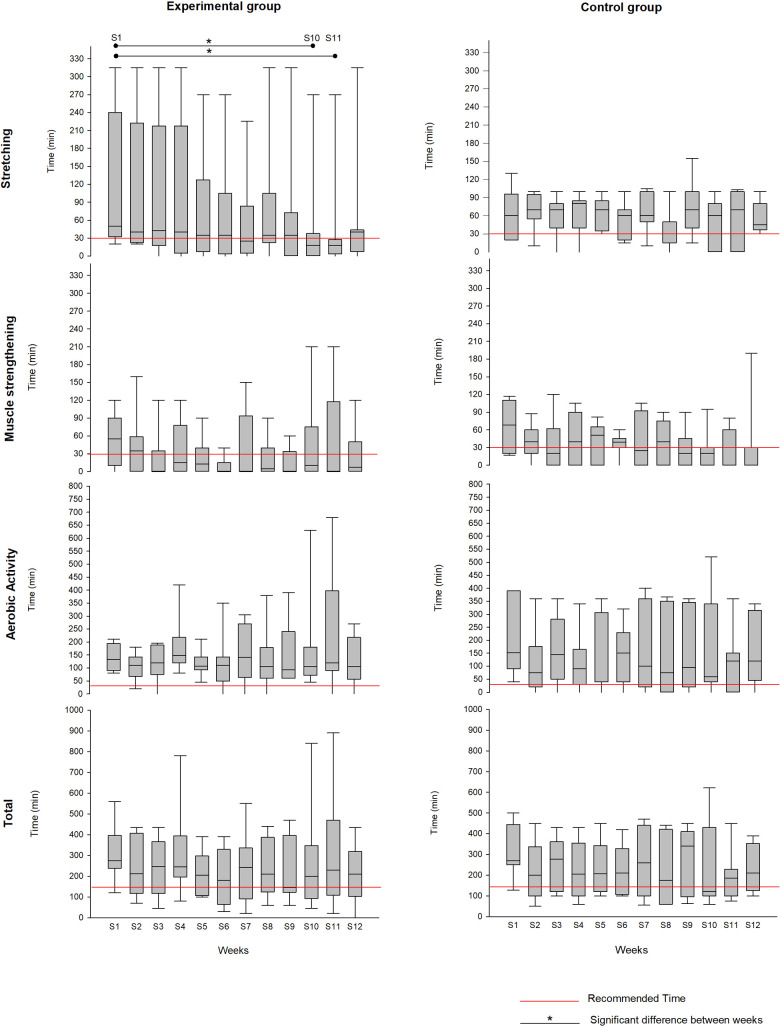
Practice (median or mean) of different types of activities, for each group, during the 12 weeks of the home programme.

Figure 2 graphically presents the one-way repeated measures ANOVA for each group and each type of activity over the 12 weeks. A significant difference between weeks 1 [median: 50 min (32.5; 240)] and 10 [median: 17.5 min (0; 37.5)], as well as 1 and 11 [median: 17.5 min (3.8; 27.5)], was found for stretching in the experimental group.

### Group comparison at T0 and T1

The comparisons of epidemiological data (age, gender, BMI) and ICF-related variables between the groups at T0 and T1 are shown in Table 2. There was a significant difference only in mean age (*p* < 0.004), with the experimental group (48.7 ± 9.6 years) having a higher mean age than the control group (37.7 ± 9.7 years). Regarding the ICF-related variables, there were no significant differences between groups at T0 or T1.

**Table 2:** Group comparison before the multidisciplinary programme (T0) and before the home-based programme (T1).

	T0		T1	
	Experimental	Control	Experimental	Control
	Mean (SD)	Mean (SD)	Mean (SD)	Mean (SD)
Patients, *n*	15	15	15	14
Age, years	**48.7 (±9.6)**	**37.7 (±9.7)**		
Gender, F/M	8/7	8/7		
BMI	22.5 [22.8–32.8]*	22.8 [19.8–29.8]*		
VAS, rest, mm	46 [27–56]*	45 [14–67]*	23 [7–38]*	13 [8.8–29]*
VAS, effort, mm	76 [61–85]*	80 [68–84]*	40 [13–58]*	28 [23.3–40.5]*
FTF, cm	23.3 (14.7)	13.3 (12.7)	10 [−11 to 14]*	3.5 [−6 to 16]*
HTB, left, cm	14 (8.9)	16.3 (10.9)	6 [0–9]*	2.5 [0–9.8]*
HTB, right, cm	16.4 (8.5)	16.1 (13.1)	6 [0–12]*	1 [0–10.5]*
Shirado, sec	36 [19–80]*	41 [30–72]*	103 [66–52.8]*	77 [52.75–129]*
Sorensen, sec	15 [10–64]*	33 [24–81]*	87.2 (58.4)	94.2 (26.1)
RDQ, 0–24	11 [6–13]	8 [6–14]*	7 [4.3–8.5]*	6 [2–7]*
Dallas				
1. Work and leisure activities, %	53.3 [46.7–66.7]*	53.3 [40–60]*	43.3 [25–56.7]*	36.7 [25–54.2]*
2. Anxiety/Depression, %	46.7 [20–60]*	33.3 [20–60]*	20 [6.7–36.7]*	16.7 [0–37.5]*
3. Sociability, %	26.7 [6.7–46.7]*	33.3 [20–53.3]*	20 [5–40]*	16.7 [0–33.3]*
FABQ-Physical, 0–18	7 [4–10]*	8 [4–12]*	3.5 [0.8–10.3]*	4 [0–5]*

BMI, body mass index; FABQ, fear avoidance belief questionnaire; FTF, finger-to-floor distance; HTB, heel-to-buttock distance; RDQ, roland disability questionnaire; SD, standard deviation; VAS, visual analogue scale.

Significant results (*p* < 0.05) are in **bold**.

*Median [Quartile 1–Quartile 3].

### Effects of the multidisciplinary programme on ICF-related variables

The two-way repeated measures ANOVA showed a significant time effect for measures of sub-pelvic mobility (fingertip-to-floor distances) and muscle endurance (Shirado and Sorensen), but no group effect or group-time interaction (Supplementary Table S1). The Wilcoxon test showed significant differences between T0 and T1 for the two groups combined, for all ICF-related variables analysed (Supplementary Table S2).

### Effects of the home-based programme on ICF-related variables

The two-way repeated measures ANOVA showed a group-time interaction for the heel-to-buttock distance on the right side, but no group or time effect (Supplementary Table S1).

Intra-group analysis with the Wilcoxon test showed a significant difference between T1 and T2 for the heel-to-buttock distance on the left side (median T1: 6 cm [0; 9]; median T2: 7.5 cm [0; 10.4]) in the experimental group. It also showed a significant difference between T1 and T2 for the scores on the Roland Disability Questionnaire (median T1: 6 points [2; 7]; median T2: 1.5 points [0; 4.5]) and the Dallas subscale “Work and leisure activities” (median T1: 36.7% [25; 54.2]; median T2: 10% [5; 28.4]) in the control group. Inter-group analysis with the Mann-Whitney test presented no significant difference between groups (Supplementary Table S3).

## Discussion

### Main findings

Our objectives were to assess the compliance with and effectiveness of a personalised home exercise programme following multidisciplinary rehabilitation. According to our findings, compliance was good for all activities in both groups. The exercise booklet and recommendations were probably enough to keep most patients motivated. On the other hand, the implementation of an individualised home exercise programme did not result in greater compliance with physical activity. Moreover, with regard to the change in time spent practising the activities over the 12 weeks at home, we noted that the experimental group significantly decreased the time spent stretching. Thus, for patients who have more difficulty maintaining regular exercise, another source of motivation may be needed. A previous study pointed out that the factors of adherence to a home-based programme in this population are highly variable (23). Some patients express the need for support and regular follow-up to stay motivated, others need more challenge, and others more feedback on their performance. Consequently, the strategies proposed to enhance adherence should be also personalised.

Of the three categories of activity, aerobic activity was the most practised, with both groups performing almost 500% of what was recommended. We did not include a specific evaluation of aerobic capacity, but it would be interesting to determine whether this amount of aerobic activity resulted in a real improvement. In any case, it is probable that it contributed to the maintenance of the ICF-related variables up to 3 months after the multidisciplinary programme.

Regarding our secondary outcomes, the multidisciplinary programme led to an improvement in all the ICF-related variables for all patients. The groups were comparable before the multidisciplinary programme (T0), and remained so after (T1), confirming this positive change for all participants. We expected these results, since all participants participated in the same programme, but it seemed important to ensure that all patients were responsive to this treatment. Only one participant left the multidisciplinary programme prematurely due to severe back pain, but we do not know if this was due to the programme itself.

For the majority of the ICF-related variables, patients in both groups were able to maintain the benefits acquired in the multidisciplinary programme at 3 months (T2). However, according to the intra-group analysis, the control group continued to improve functionally (Roland Disability Questionnaire) and in their participation in work and leisure activities (Dallas subscale 1), whereas the experimental group regressed in sub-pelvic mobility (heel-to-buttock measure). On the other hand, the inter-group analysis found no significant difference in the changes in the groups between T1 and T2. When we consider these results in relation to those on therapeutic compliance, it is interesting to note that the loss of sub-pelvic mobility in the experimental group could be explained by the decrease in stretching exercise; also, the improvements in the control group could be explained by their good exercise compliance.

### Limitations

Our assessment protocol had some limitations. First, the notion of compliance is difficult to measure because there are few, if any, validated clinical tools for CLBP. Indeed, it is difficult to objectively evaluate whether a person is doing the prescribed exercises independently if there is no third party to attest to the performance of these exercises. We chose the logbook method because it is easy to implement and provides accurate quantitative data. The limitation is that patients might deliberately falsify their results, sometimes forget to record them, or become discouraged from keeping daily records (24). This may explain why we had so much data loss at the 3-month follow-up assessment. Also, the small simple size can cause a higher chance of type 2 error, thus not allowing us to find difference between groups that could be in fact present (25). In addition, both groups had to complete their logbooks and thus receive feedback on the amount of exercise they completed each week. This feedback may have had an effect on adherence by reminding patients that they needed to exercise. Thus, the compliance of the control group could have been biased by this feedback. A third group without a logbook would have allowed us to assess the real impact of this tool. Regarding the ICF-related criteria, the heel-to-buttock distance is not the most commonly used measure in the literature, and we do not know its reliability or validity. Instead, a goniometric measurement of knee flexion in the prone position might have been more relevant (26, 27).

The other major limitation is our sample size. Of the 30 patients initially recruited, 20 returned for the final assessment, and only 15 of them completed their logbooks. This substantial loss of data greatly reduced the reliability of our results. Future research should take into account this important dropout.

Finally, our 3-month follow-up may not have been sufficient to observe a drop in compliance. According to previous studies on the implementation of an exercise programmes for CLBP sufferers, compliance drops between 3 and 6 months (28, 29). Therefore, future research should consider a longer-term follow-up of personalised home exercise programmes.

### Clinical recommendations

Based on our findings, we recommend multidisciplinary programmes for patients with CLBP. We also recommend an illustrated booklet and advice on how to continue the exercises at home. We believe that it would be appropriate to implement a longer-term follow-up to ensure that patients continue regular physical activity and, if not, to set up new strategies for therapeutic compliance. However, we cannot yet conclude on the usefulness of a personalised home exercise programme.

### Recommendations for future research

Several research directions could be explored to improve adherence. For example, it would be interesting to propose a longer-term follow-up with private-practice professionals. Patients could have once-weekly sessions with a physical activity educator or physiotherapist, in addition to their home-based programme (30, 31). It is also possible to use tele-rehabilitation (32, 33). The implementation of group physical activity *via* videoconferencing could be a form of transition after the multidisciplinary programme. This way patients would still feel supported while building the habit of exercising at home.

Future research might also focus on developing a compliance measurement tool. This tool should incorporate both quantitative and qualitative measures, with these latter being as accurate and objective as possible, without being too complex for the patients. New technologies, such as smartphones, offer interesting possibilities for tracking.

## Conclusion

The primary objective was to assess the compliance with a personalised home exercise programme after multidisciplinary rehabilitation for CLBP. The secondary objective was to evaluate the effectiveness of the multidisciplinary programme according to the relevant ICF criteria. Our results showed no difference in compliance between groups after an average of 12 weeks of home exercise. However, our remaining sample was very small at the end, which limits the generalisation of these results. The multidisciplinary programme resulted in an improvement in all our ICF criteria, and these changes were sustained over 12 weeks of exercising at home. We therefore recommend this type of programme, but we cannot yet conclude on the relevance of a personalised home exercise programme afterwards. Future studies could focus on following these patients in the longer term (after 6 months) or might target the least compliant subjects with the aim of developing personalised strategies to support them.

## Data Availability

The original contributions presented in the study are included in the article/**Supplementary Material**, further inquiries can be directed to the corresponding author/s.
