# Supplementary material for: Compliance with a personalised home exercise programme in chronic low back pain patients after a multidisciplinary programme: A pilot randomised controlled trial
Source: Front Rehabil Sci. 2022 Nov 17;3:1050157. doi: 10.3389/fresc.2022.1050157 (PMC9712950; doi:10.3389/fresc.2022.1050157)
Supplement: Supplementary file 1 [file TableS1.docx]

Table 1: Two-way repeated measures ANOVA for parametric data comparing the effect of the multidisciplinary programme (T0-T1) and the home-based programme (T1-T2) between groups

|  | Experimental | Control | Group Effect | Time Effect | Group X Time Effect |
| --- | --- | --- | --- | --- | --- |
|  | *Mean differences (SD)* | *Mean differences (SD)* | *P- Value* | *P- Value* | *P- Value* |
| T0 – T1 | | | | | |
| HTB, left, cm | -5,7 (8,8) | -9,4 (11,2) | 0,894 | **<0,001** | 0,332 |
| HTB, right, cm | -6,9 (10,5) | -8,4 (12,4) | 0,766 | **0,001** | 0,740 |
| Shirado, sec | 62,9 (48,2) | 43,4 (39,3) | 0,558 | **<0,001** | 0,245 |
| Sorensen, sec | 42,5 (42,7) | 48,8 (35,3) | 0,503 | **<0,001** | 0,762 |
| T1 – T2 | | | | | |
| HTB, right, cm | 3,5 (5,4) | -2,3 (5) | 0,168 | 0,612 | **0,023** |
| Shirado, sec | 24,1 (85,1) | 25 (59,5) | 0,246 | 0,114 | 0,851 |
| Sorensen, sec | 18 (44,2) | 32,7 (70,2) | 0,585 | 0,06 | 0,723 |
| Abbreviations: HTB, heel-to-buttock distance; SD, Standard Deviation.  Significant results (p<0.05) are in **bold** | | | | | |
