# Supplementary material for: Compliance with a personalised home exercise programme in chronic low back pain patients after a multidisciplinary programme: A pilot randomised controlled trial
Source: Front Rehabil Sci. 2022 Nov 17;3:1050157. doi: 10.3389/fresc.2022.1050157 (PMC9712950; doi:10.3389/fresc.2022.1050157)
Supplement: Supplementary file 2 [file TableS2.docx]

Table 2: Wilcoxon test for non-parametric data to evaluate the effect of the multidisciplinary programme

|  | Patients of all groups (n=30)  T0 | Patients of all groups (n=29)  T1 |
| --- | --- | --- |
|  | *Median* | *Median* |
| VAS, rest, mm | ***45,5 [20,8; 61,3]*** | ***20 [8,5; 61,3]*** |
| VAS, effort, mm | ***79 [62,8; 84,3]*** | ***34 [22,5; 52,5]*** |
| FTF, cm | ***22 [2,8; 27,5]*** | ***7 [-8; 15,5]*** |
| RDQ, 0-24 | ***10 [6; 13]*** | ***6 [2,5; 7,8]*** |
| Dallas | | |
| 1. Work and leisure activities, % | ***53,3 [40; 66,7]*** | ***40 [26,7; 53,3]*** |
| 2. Anxiety/Depression, % | ***43,4 [20; 60]*** | ***20 [6,7; 35,8]*** |
| 3. Sociability, % | ***30 [18,3; 53,3]*** | ***20 [1,7; 33,3]*** |
| FABQ-Physical, 0-18 | ***8 [4; 10]*** | ***4 [0,3; 8,3]*** |
| Abbreviations: FABQ, Fear Avoidance Belief Questionnaire; FTF, finger-to-floor distance; RDQ, Roland Disability Questionnaire; SD, Standard Deviation; VAS, Visual Analogue Scale.  Significant results (p<0.05) are in **bold**  Highly significant results (p<0.01) are in **bold** and ***italics*** | | |
