# Supplementary material for: Compliance with a personalised home exercise programme in chronic low back pain patients after a multidisciplinary programme: A pilot randomised controlled trial
Source: Front Rehabil Sci. 2022 Nov 17;3:1050157. doi: 10.3389/fresc.2022.1050157 (PMC9712950; doi:10.3389/fresc.2022.1050157)
Supplement: Supplementary file 3 [file TableS3.docx]

Table 3: Mann-Whitney and Wilcoxon tests for nonparametric data comparing the effect of the home-based programme between groups, and within each group.

|  | Mann-Whitney | | Wilcoxon | | | |
| --- | --- | --- | --- | --- | --- | --- |
|  | Experimental (n=15)  *Median of differences* | Control (n=14)  *Median of differences* | Experimental (n=15)  *Median [Quartile 1- Quartile 3]* | | Control (n=14)  *Median [Quartile 1- Quartile 3]* | |
|  | T2-T1 | T2-T1 | T1 | T2 | T1 | T2 |
| VAS, rest, mm | 2 [-7,8; 16,5] | -6 [-22,3; 5,5] | 23 [7; 38] | 19 [5; 54] | 13 [8,8; 29] | 6 [0; 15,5] |
| VAS, effort, mm | 9 [-13,5; 31] | 6 [-18; 14,5] | 40 [13; 58] | 53 [33,3; 71,3] | 28 [23,3; 40,5] | 30 [10; 40,8] |
| FTF, cm | 3 [-1; 8,3] | 2[-5,5; 3] | 10 [-11; 14] | 2 [-9; 13,5] | 3,5 [-6; 16] | -1 [-3; 10] |
| HTB, left, cm | 2,5 [0; 7,4] | 0 [-5; 2] | 6 [0; 9] | **7,5 [0; 10,4]** | 2,5 [0; 9,8] | 6 [0; 11] |
| RDQ, 0-24 | -1 [-2,3; 0,8] | -2 [-3; -1] | 7 [4,3; 8,5] | 5 [0,8; 10,8] | 6 [2; 7] | ***1,5 [0; 4,5]*** |
| Dallas |  |  |  |  |  |  |
| 1. Work and leisure activities, % | -6,7 [-14,1; 6,6] | -20 [-35; -5] | 43,3 [25; 56,7] | 38,3 [12,5; 63,3] | 36,7 [25; 54,2] | ***10 [5; 28,4]*** |
| 2. Anxiety/Depression, % | 0 [-8; 16,7] | -3,4 [-30; 3,4] | 20 [6,7; 36,7] | 23,4 [5; 58,3] | 16,7 [0; 37,5] | 0 [0; 21,7] |
| 3. Sociability, % | 0 [-10; 3,3] | -3,3 [-20; 0] | 20 [5; 40] | 16,7 [5; 21,7] | 16,65 [0; 33,3] | 3,4 [0; 20] |
| FABQ-Physical, 0-18 | 1 [-7,3; 3] | -0,5 [-2,5; 0] | 3,5 [0,8; 10,3] | 4 [2; 10,5] | 4 [0; 5] | 0 [0; 2,5] |
| Abbreviations: FABQ, Fear Avoidance Belief Questionnaire; FTF, finger-to-floor distance; HTB, heel-to-buttock distance; RDQ, Roland Disability Questionnaire; VAS, Visual Analogue Scale.  Significant results (p<0.05) are in **bold**  Highly significant results (p<0.01) are in **bold** and ***italics*** | | | | | | |
